# Mannose‐Decorated Co‐Polymer Facilitates Controlled Release of Butyrate to Accelerate Chronic Wound Healing

**DOI:** 10.1002/adhm.202300515

**Published:** 2023-08-22

**Authors:** Abigail L. Lauterbach, Anna J. Slezak, Ruyi Wang, Shijie Cao, Michal M. Raczy, Elyse A. Watkins, Carlos J. Medina Jimenez, Jeffrey A. Hubbell

**Affiliations:** ^1^ Pritzker School of Molecular Engineering University of Chicago Chicago IL 60637 USA

**Keywords:** butyrate, chronic wound healing, drug delivery, polymers, regenerative medicine

## Abstract

Butyrate is a key bacterial metabolite that plays an important and complex role in modulation of immunity and maintenance of epithelial barriers. Its translation to clinic is limited by poor bioavailability, pungent smell, and the need for high doses, and effective delivery strategies have yet to realize clinical potential. Here, a novel polymeric delivery platform for tunable and sustainable release of butyrate consisting of a methacrylamide backbone with butyryl ester or phenyl ester side chains as well as mannosyl side chains, which is also applicable to other therapeutically relevant metabolites is reported. This platform's utility in the treatment of non‐healing diabetic wounds is explored. This butyrate‐containing material modulated immune cell activation in vitro and induced striking changes in the milieu of soluble cytokine and chemokine signals present within the diabetic wound microenvironment in vivo. This novel therapy shows efficacy in the treatment of non‐healing wounds through the modulation of the soluble signals present within the wound, and importantly accommodates the critical temporal regulation associated with the wound healing process. Currently, the few therapies to address non‐healing wounds demonstrate limited efficacy. This novel platform is positioned to address this large unmet clinical need and improve the closure of otherwise non‐healing wounds.

## Graphical abstract

A novel delivery platform for the temporally controlled release of the microbial metabolite butyrate to accelerate the healing of chronic wounds is reported. Butyrate is implicated in the improvement of barrier function and is here employed to address the large clinical problem of non‐healing cutaneous wounds.

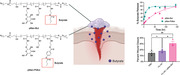

## 1 Introduction

Wound healing is a critical but complex process in which every cellular player and soluble signal is tightly controlled.^[^ ^1^ ^]^ Dysfunction in the wound is associated with many disease states and can disrupt and inhibit wound closure through perturbations in these signaling pathways and cellular interactions. It is well known that the diabetic disease state causes dysfunction in many aspects of the wound, ultimately leaving the diabetic wound chronically inflamed and unable to mount the classical tissue damage response, leaving the wound chronically open and susceptible to infection.^[^ ^2^, ^3^ ^]^ Due to this high risk of infection in non‐healing wounds, diabetic patients make up 65% of amputees in the United States.^[^ ^4^ ^]^ Despite decades of efforts to improve the outcomes of chronic wounds, few treatment strategies have shown efficacy in large‐scale, well‐controlled clinical studies, leaving clinicians with few reliable options for treatment.^[^ ^5^ ^]^ For this reason, it is of strong clinical interest to develop novel inducers of healing for chronic wounds.

Butyrate is a short‐chain fatty acid produced through bacterial fermentation of dietary fiber in the intestinal lumen. It has been proposed that butyrate production offers protection against colonic inflammation and oxidative stress and increases epithelial barrier integrity.^[^ ^6^ ^]^ While these mechanisms are not entirely understood, data suggests that butyrate exerts anti‐inflammatory effects through inhibition of NFκB signaling.^[^ ^7^, ^8^ ^]^ Separately, butyrate modulates barrier function by promoting cell migration^[^ ^9^ ^]^ and increasing production of mucins^[^ ^10^ ^]^ and heat shock proteins.^[^ ^11^, ^12^ ^]^ Clearly, butyrate plays important roles in regulating cellular function and could offer unique promise in modulating the wound environment in the case of chronic wounds, where the immune and metabolic phenotype is dysregulated.

Given the clear clinical need to alter the disrupted immune phenotype of non‐healing wounds, we hypothesized that butyrate's immunosuppressive and barrier promoting activity could be beneficial to promote an exit from the chronic, inflammatory phase of wound healing into a pro‐regenerative state.^[^ ^13^ ^]^ However, butyrate has a notoriously pungent smell, even in solution, that limits its potential as a topical therapy. Consequently, we developed a delivery platform from which butyrate is in an inactive, esterified form and is released over time via nonspecific esterases,^[^ ^14^ ^]^ with the release rate being differential based on the ester chemistry employed. The material is a co‐polymer with pendant mannose groups that increase solubility and promote receptor‐mediated endocytosis on mannose receptor‐expressing cells.^[^ ^15^, ^16^ ^]^ Here, we evaluate the in vitro and in vivo immunological bioactivity of our butyrate co‐polymers and demonstrate their promise in a murine model of diabetic wound healing.

## 2 Results

### 2.1 Differential, Sustained Release of Butyrate from Mannose Co‐Polymer

**Figure 1:**
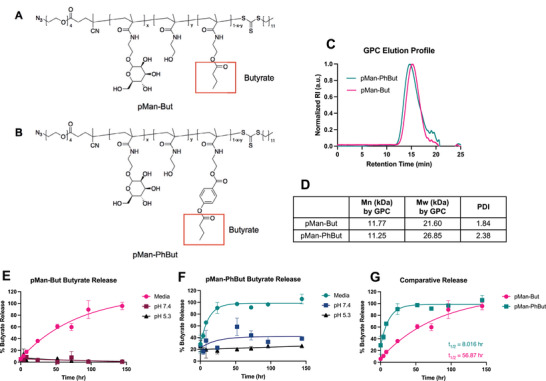
Structure and characterization of pMan‐But and pMan‐PhBut. Structures of random co‐polymers A) pMan‐But and B) pMan‐PhBut, with butyrate highlighted in a red box—the terminal carboxyl alcohol of the released butyrate is incorporated during ester hydrolysis from surrounding water, and the oxygen here is left as a free primary alcohol on the polymer chain. C) GPC elution profiles of both polymers and D) resulting molecular weight characterization as compared to polymethyl methacrylate standards. Solvent‐dependent kinetics of butyrate were measured via LC‐MS from E) pMan‐But and F) pMan‐PhBut in complete RPMI cell culture media, PBS at pH 7.4, and PBS at pH 5.3. G) Comparative release rates of butyrate from pMan‐But and pMan‐PhBut in cell culture media. Data are presented as mean +/‐ SEM. Release curves were fit based on a one‐phase decay model, and half‐lives were calculated from fit curves.

To vary release kinetics of butyrate from the co‐polymer, we synthesized methacrylamide monomers with either an aliphatic ester or a phenyl ester as described in the Experimental Section. Either of two monomers were co‐polymerized with a mannose‐functionalized methacrylamide monomer and with water‐soluble spacer monomer hydroxyethyl methacrylamide (HEMA) via RAFT polymerization to produce poly[*N*‐(2‐(α‐D‐mannose)ethyl)methacrylamide‐*stat*‐N‐(2‐butanoyloxyethyl)methacrylamide‐*stat*‐*N*‐(2‐hydroxyethyl)methacrylamide] (referred to as pMan‐But) (**Figure** **1**A) or poly[*N*‐(2‐(α‐D‐mannose)ethyl)methacrylamide‐*stat*‐*N*‐(2‐(4‐butanoyloxybenzoyloxy)ethyl)methacrylamide‐*stat*‐*N*‐(2‐hydroxyethyl)methacrylamide] (referred to as pMan‐PhBut) (Figure 1B). Monomers were incorporated at a ratio of mannose:HEMA:butyrate of 2:7:4 to balance water solubility and high butyrate payload. Gel permeation chromatography (GPC) analysis of both polymers revealed a single, broad elution profile (Figure 1C) corresponding to a similar number‐averaged molecular weight around 11 kDa (Figure 1D). We also characterized the polymers via ^1^H‐NMR (Figure S1A,B, Supporting Information). With the resulting materials, we analyzed butyrate release kinetics in a variety of solutions. We found that negligible butyrate was released from either polymer in phosphate‐buffered saline, regardless of pH within the physiological range (Figure 1E,F). It was only in complete media, which contained 10% v/v fetal bovine serum (FBS), that butyrate released from the polymer backbone, which could be attributed to esterase‐like activity of albumin or the presence of esterase enzymes.^[^ ^17^, ^18^ ^]^ When directly comparing the two polymers, we found that butyrate released faster from pMan‐PhBut than from pMan‐But, with half‐lives of 8.0 and 56.9 h, respectively (Figure 1G). We also found that total butyrate released was equivalent to a mass ratio of approximately 10% w/w.

### 2.2 pMan‐But and pMan‐PhBut Modulate Inflammation In Vitro

**Figure 2:**
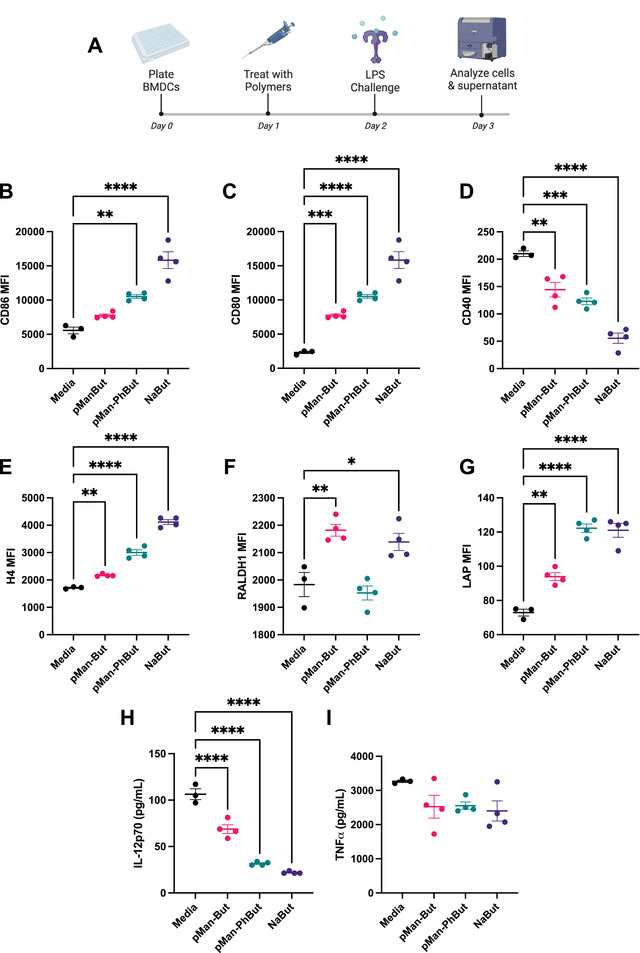
In vitro characterization of butyrate polymer bioactivity. A) A schematic of the experimental timeline. Murine BMDCs were treated with pMan‐But, pMan‐PhBut, or unmodified sodium butyrate (NaBut) at a concentration of 1 mm butyrate equivalent. After 18 h, endotoxic lipopolysaccharide (LPS) was added at 100 ng mL^−1^, and cells and supernatant were analyzed 24 h later. Flow cytometric analysis of the cells themselves included quantification of B) CD86, C) CD80, D) CD40, E) H4, F) RALDH1, and G) LAP expression. Analysis of the supernatant via ELISA allowed for quantification of secreted H) IL‐12p70 and I) TNFα. Statistical analysis was performed using ordinary one‐way analysis of variance with multiple comparisons with respect to media control. *p < 0.05, **p < 0.01, ***p < 0.001, ****p < 0.0001, and ns, not significant.

After confirming our polymer itself is not toxic to RAW264.7 macrophage‐like cells in vitro (Figure S2A, Supporting Information), we moved to assess the bioactivity of our constructs in comparison to unmodified sodium butyrate (NaBut) using an in vitro model of prophylactic immune suppression in response to lipopolysaccharide (LPS) stimulation using murine bone‐marrow derived dendritic cells (BMDCs) (**Figure** **2**A). We again confirmed that doses used were non‐toxic to cells by measuring cell viability (Figure S2B, Supporting Information). In response to pre‐treatment of BMDCs by the butyrate‐containing co‐polymers, we broadly observed changes in cell‐surface receptor expression and suppression of pro‐inflammatory signaling similar to those mediated by unmodified NaBut. Levels of both T cell co‐stimulatory factors CD80 and CD86 were upregulated as compared to cells without butyrate pre‐treatment (Figure 2B,C), which is consistent with other reports of butyrate bioactivity in vitro.^[^ ^19^ ^]^ Levels of CD40, a marker of activated DCs, were decreased with all types of butyrate pre‐treatment (Figure 2D); precise modulation of CD40‐CD40L interactions has been implicated at various stages of wound healing.^[^ ^20^, ^21^ ^]^ Next, we observed a significant upregulation in histone H4 staining, indicating a characteristic inhibition of histone deacetylase 4 (HDAC4) (Figure 2E),^[^ ^22^, ^23^ ^]^ which may also have implications on keratinocyte differentiation.^[^ ^24^ ^]^ Interestingly, we saw an increase in retinaldehyde dehydrogenase 1 (RALDH1) (Figure 2F), which is activated by butyrate downstream of both GPCR109A activation and HDAC inhibition,^[^ ^25^ ^]^ in only pMan‐But and NaBut, but not in pMan‐PhBut. These markers together indicate phenotypic butyrate bioactivity of our polymers and potential for pro‐regenerative signaling in the wound.

As for cytokine profiles, the latency‐associated peptide (LAP) of TGF‐β is upregulated (Figure 2G), corresponding to an increase in precursor TGF‐β that could be activated to yield the anti‐inflammatory cytokine by integrin signaling.^[^ ^26^ ^]^ LPS‐induced secretion of the pro‐inflammatory cytokine IL‐12p70 was markedly reduced with all butyrate pre‐treatments (Figure 2H), however, we did not observe a change in TNFα production (Figure 2I). We further characterized BMDC and RAW‐264.7 phenotypes in response to pMan‐But stimulation (Figures S3 and S4, Supporting Information). Together, these results suggest that pMan‐But and pMan‐PhBut modulate cellular immune phenotypes similarly to NaBut in a way that could be beneficial for wound healing.

### 2.3 pMan‐But But Not pMan‐PhBut Modulate Soluble Signals In Vivo

**Figure 3:**
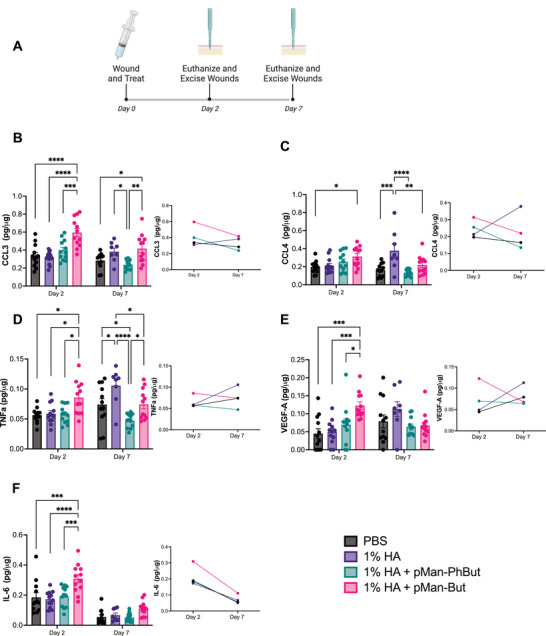
In vivo characterization of butyrate polymer immune milieu modulation. A) A schematic of the experimental timeline. Male db/db mice had four 6 mm wounds excised from dorsal cutaneous tissue and were subsequently treated with PBS, 1% HA, 1% HA + pMan‐But, or 1% HA + pMan‐PhBut at a concentration of 12.5 mg mL^−1^ butyrate equivalent. After 2 or 7 days mice were sacrificed and their wounds harvested and homogenized. Multiplexed soluble signal analysis of the homogenate was collected quantifying wound content of B) CCL3, C) CCL4, D) TNFα, E) VEGF‐A, and F) IL‐6. Statistical analysis was performed using ordinary two‐way analysis of variance with Tukey's multiple comparison test against all groups. *p < 0.05, **p < 0.01, ***p < 0.001, ****p < 0.0001, and ns, not significant.

After demonstrating that our constructs induced changes in immune activation in vitro, we moved toward assessing the changes in soluble signals induced through treatment in vivo. We utilized the type 2 diabetic (db/db) excisional wound murine model, treating the wounds at the time of incision and measuring cytokines and chemokines after 2 or 7 days (**Figure** **3**A). To provide viscosity for the purpose of retention of the polymers at the site of application, they were dissolved in a 1% hyaluronic acid (HA) carrier, and the carrier was studied as a treatment as well. Importantly, HA is permeable to metabolites such as butyrate.^[^ ^27^, ^28^ ^]^ Broadly, we saw that pMan‐But induced significant pro‐regenerative shifts in the milieu of the wound microenvironment. Levels of the chemokines CCL3, a macrophage chemotactic recruitment factor, were significantly higher after treatment with pMan‐But when compared to all other treatment groups 2 days post treatment, but decreased by day 7 (Figure 3B). The temporal profile indicates a healthy recruitment and phagocytic response with subsequent resolution, which is reported to be dysregulated in the untreated diabetic wound.^[^ ^29^, ^30^ ^]^ A similar trend can be seen in the chemokine CCL4 profile, another macrophage chemotactic factor,^[^ ^31^ ^]^ which showed an initial increase in expression followed by its gradual decrease (Figure 3C). Treatment of the wound with pMan‐But also induced early elevation of TNFα followed by a slight decrease in expression over time, while other treatment groups had the opposite trend with gradual increases in TNFα levels by day 7 (Figure 3D). Early induction of TNFα has been shown to be a crucial component of complete closure of a cutaneous wound.^[^ ^32^ ^]^

Angiogenic factors are known to play a critical role in wound healing and are found to be diminished in the chronic wound.^[^ ^33^ ^]^ Treatment with pMan‐But induced a strong increase in VEGF‐A expression, the most prominent angiogenic factor, on day 2. This strong induction was resolved by day 7, while all other groups displayed the opposite trend (Figure 3E). This striking temporal trend indicates a transition between wound healing phases, where blood vasculature is no longer being produced but rather pruned and substantiated.^[^ ^34^ ^]^ Lastly, pMan‐But, but not pMan‐PhBut, induced a profound increase in the expression of IL‐6 on day 2 that was resolved by day 7 (Figure 3F). IL‐6 has been implicated in the migration of fibroblasts and keratinocytes to sites of injury and promotes deposition of the extracellular matrix components that are required for re‐epithelization.^[^ ^35^, ^36^ ^]^ These results suggest that pMan‐But, but not pMan‐PhBut, administered in 1% HA favorably modulates the soluble signal profile in the wound microenvironment to a milieu more conducive to regeneration of the wound. In the in vivo experiments, we did not study free NaBut administered in 1% HA since it is not a relevant formulation due to its unacceptable odor.

### 2.4 pMan‐But Treatment Accelerates Wound Healing in Type 2 Diabetic Murine Model

**Figure 4:**
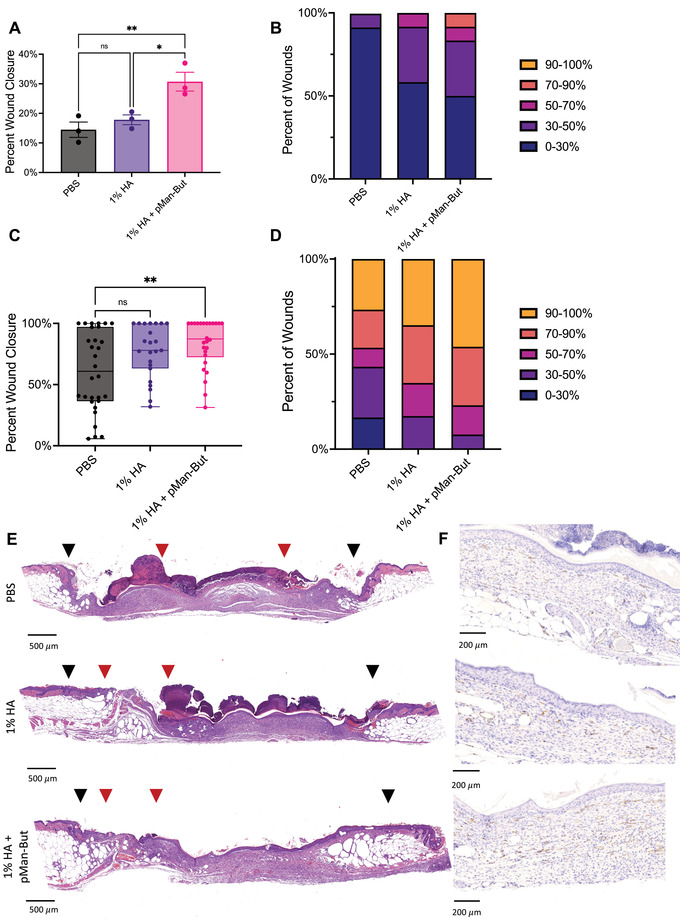
In vivo wound closure efficacy of butyrate polymer. Male db/db mice had four 6 mm wounds excised from dorsal cutaneous tissue and were subsequently treated with PBS, 1% HA, or 1% HA + pMan‐But at a concentration of 12.5 mg mL^−1^ butyrate equivalent. After 7 or 11 days mice were sacrificed and their wounds excised for histological analysis. A) Wound closure percentage after 7 days. B) Percent of wounds within each bin of closure after 7 days. C) Percent wound closure after 11 days, experiment was repeated a total of two times and pooled. D) Percent of wounds within each bin of closure after 11 days. E) Representative H&E images after 11 days. The black arrowheads indicate the margin of the original wound and red arrowheads indicate the tips of epithelium tongue. F) Representative *CD*31^+^ IHC images after 11 days. Statistical analysis was performed using a) ordinary one‐way analysis of variance with Tukey's multiple comparison test against all groups or b) Kruskal–Wallis test with Dunn's multiple comparison with respect to PBS control. *p < 0.05, **p < 0.01, and ns, not significant.

Due to the favorable shift we saw in the soluble signals present in the wound microenvironment after treatment with pMan‐But, we tested its efficacy in a db/db cutaneous excisional model. To better understand the material properties of the therapeutically applied gels, we performed rheometric analysis, which indicated similar physical properties between the carrier and the full therapy, the properties of which have been detailed elsewhere^[^ ^37^ ^]^ (Figure S5, Supporting Information). After the wounds were created in the dorsal cutaneous tissue, they were treated with either PBS, 1% HA (carrier), or pMan‐But in 1% HA carrier. After 7 days, pMan‐But had significantly improved healing when compared to both the PBS and carrier controls (**Figure** **4**A,B). This 7 day time point depicted an acceleration of wound closure after pMan‐But treatment, but a majority of the wound remained open. We then extended the length of time allowed for healing. After 11 days, the pMan‐But‐treated wounds showed significantly more healing compared to the PBS treatment group (Figure 4C,D), whereas the carrier group did not, although pMan‐But was not statistically different from the carrier control. We also took photographs depicting healing at endpoint at both days 7 and 11 (Figure S6, Supporting Information). Still, only pMan‐But in 1% HA showed a statistically significant increase in closure relative to the PBS‐treated group. It should also be noted that a molecular weight‐matched HEMA‐only control polymer, without mannose and butyrate, showed a hindrance to closure of these wounds at day 11 (Figure S7, Supporting Information), indicating the relevance of our functional co‐monomers. Representative H&E images (Figure 4E) visualize the improved re‐epithelialization after pMan‐But treatment at the 11 day time point. We also visualized blood vessel development indicated by CD31^+^ staining via immunohistochemistry (IHC), which suggested that pMan‐But treatment induces potent vasculature formation (Figure 4F). These results taken together indicate that pMan‐But can accelerate the healing of chronic type 2 diabetic wounds.

## 3 Discussion

The results laid out above demonstrate a platform material that is capable of dynamically delivering its payload, in this context butyrate, in a manner that is targeted at cells expressing the mannose receptor for ligand scavenging. The different release kinetics of the two constructs allow for a tunable delivery. In a prophylactic context, that is, preventing response to an inflammatory signal, both pMan‐But and pMan‐PhBut are capable of preventing signature markers of inflammation on LPS‐stimulated BMDCs. For wound healing, a slower release of butyrate was thought to be beneficial as to not blunt the important spike in inflammation that facilitates the clearance of debris in the wound.^[^ ^38^ ^]^ The initial peak of the cytokine and chemokine signals seen, followed by their gradual decrease by day 7 suggests a rebalancing of the milieu present in the wound microenvironment after treatment with pMan‐But. This result is corroborated with the enhanced morphological healing seen after pMan‐But treatment. These results taken together depict a versatile and novel polymer‐based approach to treating chronic wounds.

The pMan‐But and pMan‐PhBut polymers are implementations of a broader platform that could be extended to the delivery of other interesting small molecules and metabolites for wound healing.^[^ ^39^, ^40^, ^41^, ^42^ ^]^ We demonstrate an implementation of the material using both an aliphatic hydroxyl butyric ester and a phenyl butyric ester, which have differing release kinetics. Because the wound healing process is so dynamic, the exact timing of therapeutic release must be considered for each drug delivered.^[^ ^43^ ^]^ Furthermore, as mentioned above, the pendant mannose groups serve to enhance solubility and promote internalization, but could further promote an immunosuppressive, anti‐inflammatory environment via mannose receptor cross‐linking.^[^ ^44^ ^]^ Finally, the high molecular weight polymers, relative to small molecules like butyrate, increase viscosity and thus promote hydration in the wound.^[^ ^45^ ^]^

The therapeutic potential of butyrate is especially promising due to its established role in maintaining barrier integrity in the gut.^[^ ^46^, ^47^ ^]^ To date, most therapeutic efforts using butyrate have involved delivery to the gut to modulate disease states therein, including inflammatory bowel disease,^[^ ^48^ ^]^ Crohn's disease,^[^ ^49^ ^]^ diabetic inflammation,^[^ ^50^ ^]^ and even colorectal cancer.^[^ ^46^ ^]^ Butyrate's topical administration has been considered and attempted in various murine wound models,^[^ ^51^, ^52^, ^53^, ^54^ ^]^ but its translational potential is limited due to its particularly foul smell^[^ ^55^ ^]^ and common need for co‐delivery with another therapeutic agent. However, these accounts point to key mechanistic steps in butyrate's therapeutic efficacy, including inhibition of histone deacetylase, NLRP3 inflammasome, and pro‐inflammatory cytokine production.^[^ ^56^ ^]^

There have been many attempts to solve the large clinical problem of non‐healing diabetic wounds with few clinically impactful outcomes, leaving the current standard of care to rely primarily on physical measures such as surgical debridement and wound offloading.^[^ ^2^, ^57^, ^58^ ^]^ Outside of these methods, the many attempts made to improve chronic wound healing outcomes can be categorized into three broad approaches. First, dressings and hydrogels which act to keep the wound clean and moist.^[^ ^2^ ^]^ These dressings cover a wide array of compositions, but only provide moisture regulation and in some cases antimicrobial activity.^[^ ^59^ ^]^ Even with improvements made in the field, there remains to be seen high‐quality evidence that suggests any significant differences in wound healing outcomes when comparing various types of dressing.^[^ ^5^, ^60^, ^61^ ^]^ Second, delivery of growth factors and cytokines which aim to modulate the wound microenvironment. This approach has shown some promise, with the clinical approval of Becaplermin, a topically applied PDGF‐BB‐containing gel.^[^ ^62^ ^]^ Many other growth factors have been tested, with limited clinical success due to both their short half‐life and their widespread effects if introduced into systemic circulation.^[^ ^63^, ^64^ ^]^ The pleiotropic nature of protein‐based therapeutics is a clear pitfall and proves to be a continued difficulty when ensuring their targeted effects, though engineering attempts have been made to counter such unwanted activity.^[^ ^65^, ^66^, ^67^, ^68^ ^]^ Third, cell‐based therapies attempt to repopulate the wound with cells directly involved in healing. This approach has shown some small‐scale success with little consensus on optimal cell types.^[^ ^69^ ^]^ However, regardless of efficacy, cell‐based treatments are troubled with the problem of scale‐up, making this approach translationally challenging within the current production workflow and its constraints.^[^ ^70^, ^71^ ^]^ These approaches differ from our work because our platform, which allows the controlled local release of butyrate, circumvent many of the aforementioned limitations of these strategies.

Our data clearly demonstrate induction of immunological chemokines and cytokines thought to be beneficial for wound healing. An additional explanation for the improved healing could be HIF‐1α induction, which is closely tied to improved wound healing outcomes.^[^ ^39^, ^72^ ^]^ It has been established that butyrate can induce the expression of HIF‐1α.^[^ ^73^, ^74^ ^]^ Further, our data demonstrates increased VEGF‐A expression, a downstream factor induced through HIF‐1α activity, following pMan‐But treatment.^[^ ^75^, ^76^ ^]^ Another mechanistic explanation could be butyrate's direct activity on keratinocytes, the main cellular population responsible for re‐epithelization.^[^ ^77^ ^]^ Butyrate has been shown to induce keratinocyte differentiation and improved wound closure in vitro.^[^ ^78^, ^79^ ^]^ Furthermore, it has recently been shown that butyrate promotes and accelerates the differentiation of epidermal keratinocytes by directly altering their metabolism, enhancing skin barrier function.^[^ ^80^ ^]^ pMan‐But treatment may induce keratinocyte differentiation and migration through a butyrate‐mediated metabolic shift, ultimately improving wound closure.

In conclusion, we have demonstrated the utility of a mannose‐decorated co‐polymer for the localized and sustained release of butyrate to the diabetic wound microenvironment. The controlled release of our butyrate delivery form enabled the rebalancing of the soluble cytokine and chemokine signals present and subsequently promoted the re‐epithelialization of the wound in a type 2 diabetes model of delayed wound healing. Our novel and versatile constructs have the potential to fill a clinical gap for the treatment of chronic wounds, a pressing clinical need.

## 4 Experimental Section

Unless otherwise stated, chemicals were reagent grade and purchased from Sigma–Aldrich. All NMR spectra were collected on a Bruker Avance‐II 400 MHz NMR and analyzed with MnovaNMR (Mestrelab). Mannose monomer and azide‐terminated RAFT chain transfer agent were synthesized as previously described.^[^ ^16^ ^]^ Full synthesis schema with structures are provided in the Supporting Information.

### Small Molecule Synthesis: *N*‐(2‐butanoyloxyethyl) Methacrylamide (BMA)

*N*‐(2‐hydroxyethyl) methacrylamide (3.30 mL, 25.6 mmol, 1.0 eq), triethylamine (7.15 mL, 251.2 mmol, 2.0 eq.), and 50 mL DCM were added into a 250 mL flask. After the reaction system was cooled down by an ice bath, butyric anhydride (5.00 mL, 30.7 mmol, 1.2 eq) was added dropwise under the protection of nitrogen and was allowed to react overnight. The reaction mixture was filtered and washed sequentially by ammonium chloride solution, sodium bicarbonate solution, then water. After drying by anhydrous magnesium sulfate, the organic layer was concentrated by rotary evaporation, pure product was isolated on a silica column using DCM/MeOH (MeOH fraction v/v from 0% to 5%). The product was obtained as pale yellow oil (4.56 g, 89.6%). MS(ESI). C_10_H_17_NO_3_, *m*/*z* calculated for [M+H]^+^: 199.12, found: 199.1. ^1^H‐NMR (400 MHz, d‐chloroform) δ 0.95 (t, 3H), 1.66 (m, 2H), 1.97 (s, 3H), 2.32 (t, 2H), 3.59 (dt, 2H), 4.23 (t, 2H), 5.35 (s, 1H), 5.71 (s, 1H), and 6.19 (br s, 1H).

### Small Molecule Synthesis: 4‐Butanoyloxybenzoic Acid

4‐Hydroxybenzoic acid (1 g, 7.2 mmol, 1 eq) and triethylamine (4.04 mL, 28.8 mmol, 4 eq) were dissolved in dry DCM. To that solution, butanoic anhydride (1.42 mL, 8.6 mmol, 1.2 eq) was added. After 8 h, the solvent was removed by rotary evaporation and the crude product was purified on a silica column using DCM/MeOH (MeOH fraction v/v 0% to 2%). The product was white solid (0.92 g, yield 61%). MS (ESI). C_11_H_12_O_4_, *m*/*z* calculated for [M+H]^+^: 208.07, found: 208.0. ^1^H‐NMR (400 MHz, d‐chloroform) δ 8.12 (d, 2H), 7.16 (d, 2H), 2.56 (t, 2H), 1.82 (m, 2H), and 1.05 (t, 3H).

### Small Molecule Synthesis: *N*‐[2‐(4‐butanoyloxybenzoyloxy)ethyl] Methacrylamide (PhBMA)

Synthesized 4‐butanoyloxybenzoic acid (0.67 g, 3.2 mmol, 1.0 eq), *N*‐(2‐hydroxyethyl) methacrylamide (HEMA, 0.62 g, 4.8 mmol, 1.5 eq), and DCC (0.99 g, 4.8 mmol, 1.5 eq) were dissolved in dry DCM. The reaction mixture was stirred at 0 °C for 30 min. After that time, 4‐dimethylaminopyridine (19 mg, 0.15 mmol, 0.05 eq) dissolved in dry DCM was added to the reaction mixture dropwise. The reaction was allowed to rise to room temperature overnight. The reaction mixture was filtered and solvent was removed by rotary evaporation. The crude product was purified on a silica column using ethyl acetate:hexanes 1:1 v/v. The product was white solid (0.98 g, yield 96%). MS (ESI). C_17_H_21_NO_5_, *m*/*z* calculated for [M+H]^+^: 319.14, found: 319.1. ^1^H‐NMR (400 MHz, d‐chloroform) δ 8.07 (d, 2H), 7.18 (d, 2H), 6.24 (br, 1H), 5.71 (s, 1H), 5.35 (s, 1H), 4.48 (m, 2H), 3.72 (m, 2H), 2.57 (t, 2H), 1.97 (s, 3H), 1.79 (m, 2H), and 1.05 (t, 3H).

### Polymer Synthesis and Characterization

The polymerizations of pMan‐But or pMan‐PhBut were similar as described in a previous paper regarding other side‐chain glycopolymers.^[^ ^16^ ^]^ Briefly, mannose monomer (300 mg, 1.03 mmol), HEMA (Combi Blocks, 500 mg, 3.50 mmol), and butyrate monomer (for pMan‐But: BMA, 400 mg, 2.00 mmol; for pMan‐PhBut: PhBMA, 400 mg, 1.26 mmol) were dissolved in dry DMF in a Schlenk tube. To that solution, azide CTA (30 mg, 0.05 mmol) and free radical initiator AIBN (2 mg, 0.01 mmol) were added. The reaction vessel was degassed via four freeze–pump–thaw cycles and then heated to 70 °C to initiate polymerization. After 14 h, the reaction vessel was immersed in liquid nitrogen to stop the reaction. The polymer was precipitated in cold acetone three times. The final product was dried under reduced pressure. The product was a white powder (360 mg, yield 72%). The polymer was characterized by ^1^H‐NMR and GPC.

GPC characterizations of pMan‐But or pMan‐PhBut were performed on Tosoh EcoSEC size exclusion chromatography system using Tosoh SuperAW3000 column. Samples were dissolved in eluent DMF with 0.01 M LiBr at 1 mg mL^−1^. The temperature of the column was set to 50 °C. Refractive index (RI) detector was used to detect polymers. Number‐averaged molecular weight, weight‐averaged molecular weight, and polydispersity index were determined via the column calibration using PMMA as standards.

Both the pMan‐But and pMan‐PhBut used in the biological studies had a number average molecular weight of 11 kDa. The butyrate content of pMan‐But or pMan–PhBut was determined by LC‐MS/MS after the complete hydrolysis catalyzed by NaOH.

### Butyrate Release Kinetic Characterization: Sample Preparation

Samples were prepared and derivatized as described elsewhere.^[^ ^81^ ^]^ Briefly, pMan‐But or pMan‐PhBut was dissolved in RPMI complete cell culture media (pH 7.4), PBS buffer, or pH 5.3 acetate buffer at a concentration of 10 mg mL^−1^. 20 µL of the solution was transferred into 500 µL of water:acetonitrile 1:1 v/v at 0, 4, 8, 24, 52, 72, 96, and 144 h. The sample was centrifuged using Amicon Ultra (Merck, 3 kDa molecular mass cutoff) at 13,000 × *g* for 15 min, to remove polymers. The filtrate was stored at ‐80 °C before derivatization.

### Butyrate Release Kinetic Characterization: Sample Derivatization

3‐nitrophenylhydrazine (NPH) stock solution was prepared at 0.02 m in water:acetonitrile 1:1 v/v. EDC stock solution was prepared at 0.25 M in water:acetonitrile 1:1 v/v. 4‐Methylvaleric acid was added as internal standard. Samples were mixed with NPH stock and EDC stock at 1:1:1 ratio by volume. The mixture was placed in a heating block at 60 °C for 30 min. Samples were transferred into HPLC vials and stored at 4 °C before analysis.

### Butyrate Release Kinetic Characterization: LC Conditions

The instrument used for quantification of butyrate was Agilent 1290 UHPLC. The column used was Thermo Scientific C18 4.6 × 50 mm, 1.8 m particle size, at room temperature. Mobile phase A: water with 0.1% v/v formic acid. Mobile phase B: acetonitrile with 0.1% v/v formic acid. The injection volume was 5.0 µL and the flow rate was 0.5 mL min^−1^. Gradient of solvent: 15% mobile phase B at 0.0 min; 100% mobile phase B at 3.5 min; 100% mobile phase B at 6.0 min; and 15% mobile phase B at 6.5 min.

### Butyrate Release Kinetic Characterization: ESI‐MS/MS Method

The instrument used to detect butyrate was Agilent 6460 Triple Quad MS‐MS. Both derivatized butyrate‐NPH and 4‐methylvaleric‐NPH were detected in negative mode. The MS conditions were optimized on pure butyrate‐NPH or 4‐methylvaleric‐NPH at 1 mM. The fragment voltage was 135 V and collision energy was set to 18 V. Multiple reaction monitoring (MRM) of 222 ← 137 was assigned to butyrate, and MRM of 250 ← 137 was assigned to 4‐methylvaleric acid as internal standard. The ratio between MRM of butyrate and 4‐methylvaleric acid was used to quantify the concentration of butyrate. For butyrate releasing study, the results were plotted by Prism software (GraphPad) and fitted by exponential plateau model to calculate first‐order reaction constant.

### In Vitro Bioactivity Characterization

Murine BMDCs were prepared from C57Bl/6 mice (Charles River) as previously described.^[^ ^82^ ^]^ Day 9 BMDCs were seeded at a density of 200,000 cells per well in a round bottom 96‐well plate in IMDM with 10% FBS and 2% penicillin/streptomycin and incubated at 37 °C. After 1 day, cells were treated with pMan‐But, pMan‐PhBut, or unmodified sodium butyrate (NaBut) at a concentration of 1 mM butyrate equivalent (*n* = 4). After 18 h, LPS was added to a final concentration on 100 ng/mL. 24 h after LPS stimulation, cells and supernatant were analyzed. ELISA kits were used for IL‐12p70 (Invitrogen #88‐7121‐88) and TNFα (Invitrogen #88‐7324‐88) quantification.

Prior to flow cytometry, anti‐RALDH1 (ALDH1A1, Invitrogen #PIPA511537, Polyclonal) and anti‐Histone 4 (H4, Abcam #ab177790, clone EPR16606) antibodies were conjugated to fluorophores using Alexa Fluor antibody labeling kits (A20181) according to manufacturer's protocol. Other antibodies used included PE‐Cy7 conjugated anti‐LAP (Fisher Scientific #50‐112‐3461, clone TW7‐16B4), PerCP‐Cy5.5 conjugated anti‐CD40 (BioLegend #124623, clone 3/23), BUV737 conjugated anti‐CD80 (BD #612773, clone 16‐10A1), and APC‐eFluor780 conjugated anti‐CD86 (Invitrogen #47‐0862‐82, clone GL1). BMDCs were stained for flow cytometry. Cells were washed once in PBS and stained with LD Violet (Invitrogen #L23105) (1:500) and Fc‐block (1:200) for 15 min at 4 °C. Cells were washed in PBS + 2% FBS and surface stain was added in PBS + 2% FBS for 30 min at 4 °C. Cells were washed, fixed, and permeabilized with Foxp3 transcription factor kit (eBioscience #00‐5521‐00). Intracellular antibodies, including H4 and RALDH1, were added in permeabilization buffer for 1 h at RT. Cells were washed per manufacturer's protocol and resuspended in PBS + 2% FBS and collected on a BD LSRFortessa, with data analysis in FlowJo.

### Mouse Skin Chronic Wound Healing Model

Male C57BLKS/J‐m/Lepr db (db/db) 8–10‐week‐old mice (The Jackson Laboratory) were used. Their backs were shaved and cleaned and four full‐thickness punch‐biopsy wounds (6 mm diameter) were created in each mouse. Each wound was splinted open using a silicone ring (inner diameter 8 mm, outer diameter 12 mm) to prevent contraction. Immediately following wound induction, PBS, 1% HA, or 1% HA + pMan‐But were topically applied to the wounds. The butyrate constructs contained 10% w/w butyrate equivalent. The wounds were covered with adaptic dressing and sealed with adhesive film. Mice were single caged after the wounding surgery. After 7 and 11 days, mice were euthanized, and the skin wounds were carefully excised for histological analysis. All experiments using mice received approval from the Institutional Animal Use Committee of the University of Chicago under ACUP 72450. The animals' care was in accordance with institutional guidelines. The 11 day time point experiment was replicated a total of two times for a final sample size of PBS (*n* = 30), 1% HA (*n* = 23), pMan‐But (*n* = 26).

### Cytokine Profile in Wound Tissue

Male C57BLKS/J‐m/Lepr db (db/db) 8–10‐week‐old mice (The Jackson Laboratory) were used. Skin wounds were treated with formulations as described above. After 2 and 7 days, the wounded skin was removed as described above and transferred to T‐Per Solution (Thermo Fisher) in Lysing Matrix D containing tubes (MP Biomedical) and homogenized (MP Biomedical). Then, the solution was centrifuged, and the supernatant was retained for analysis using LegendPlex Mouse Cytokine Release Syndrome Multiplex Kit (BioLegend) carried out according to manufacturer's instruction. Cytokine values were normalized to total protein content per BCA assay (Thermo Fisher).

### Histomorphometric Analysis of Wound Sections

Wounds were fixed overnight in 2% PFA and cut down the center into two and embedded into paraffin for histological analysis on 5 µm serial sections. The extent of re‐epithelization was measured blindly by histomorphometric analysis of tissue sections (H&E stain) using QuPath software (University of Edinburgh). For analysis of re‐epithelization, the distance that the epithelium had traveled across the wound was measured; the muscle edges of the panniculus carnosus were used as an indicator for the wound boundary; and re‐epithelization was calculated as the percentage of the distance between the edges of the panniculus carnosus muscle. Wound sections damaged during processing as visualized through histology were excluded before unblinding.

### Statistical Analysis

Any details regarding normalization of data is discussed in the respective figure caption or figure caption. No evaluation of outliers was preformed. All data are presented as mean +/‐ SEM. Statistical methods were not used to predetermine the necessary sample size, rather sample sizes were chosen based on estimates from pilot experiments and previously published results such that appropriate statistical tests could yield significant results. Sample sizes and tests used for each study are listed in respective figure captions. GraphPad Prism was used for statistical analysis and data presentation.

## Conflict of Interest

J.A.H., A.L.L., A.J.S., R.W., and M.M.R. are inventors on a provisional patent application on this technology. All other authors declare no conflict of interest.

## Author Contributions

A.L.L. and A.J.S. contributed equally to this work. A.L.L., A.J.S., R.W., and J.A.H. designed the project. R.W., A.J.S., M.M.R. synthesized materials. A.L.L., A.J.S., E.A.W., C.J.M.J., and S.C. performed the experiments. A.L.L., A.J.S., R.W., M.M.R., E.A.W and J.A.H. analyzed the data. A.L.L., A.J.S., and J.A.H. wrote the manuscript. All authors edited and approved the final manuscript.

## Supporting information

Supporting Information

## Data Availability

The data that support the findings of this study are available from the corresponding author upon reasonable request.
