# Supplementary material for: Mannose‐Decorated Co‐Polymer Facilitates Controlled Release of Butyrate to Accelerate Chronic Wound Healing
Source: Adv Healthc Mater. 2023 Aug 22;12(26):2300515. doi: 10.1002/adhm.202300515 (PMC11468131; doi:10.1002/adhm.202300515)
Supplement: Supplementary file 1 — Supporting Information [file ADHM-12-2300515-s001.pdf]

ADVANCED HEALTHCARE MATERIALS

Supporting Information

for *Adv. Healthcare Mater.*, DOI 10.1002/adhm.202300515

Mannose-Decorated Co-Polymer Facilitates Controlled Release of Butyrate to Accelerate Chronic Wound Healing

*Abigail L. Lauterbach, Anna J. Slezak, Ruyi Wang, Shijie Cao, Michal M. Raczy, Elyse A. Watkins, Carlos J. Medina Jimenez and Jeffrey A. Hubbell**

Supplementary Information for:

Mannose-Decorated Co-Polymer Facilitates Controlled Release of Butyrate to Accelerate Chronic Wound Healing

Abigail L. Lauterbach,[†,‡] Anna J. Slezak,[†,‡] Ruyi Wang,[†] Shijie Cao,[†] Michal M. Raczy,[†] Elyse A. Watkins,[†] Carlos M. Jimenez,[†] Matthew Tirrell,[†] and Jeffrey A. Hubbell[*,†]

†*Pritzker School of Molecular Engineering, University of Chicago, Chicago, IL, USA*

‡*These authors contributed equally*

A

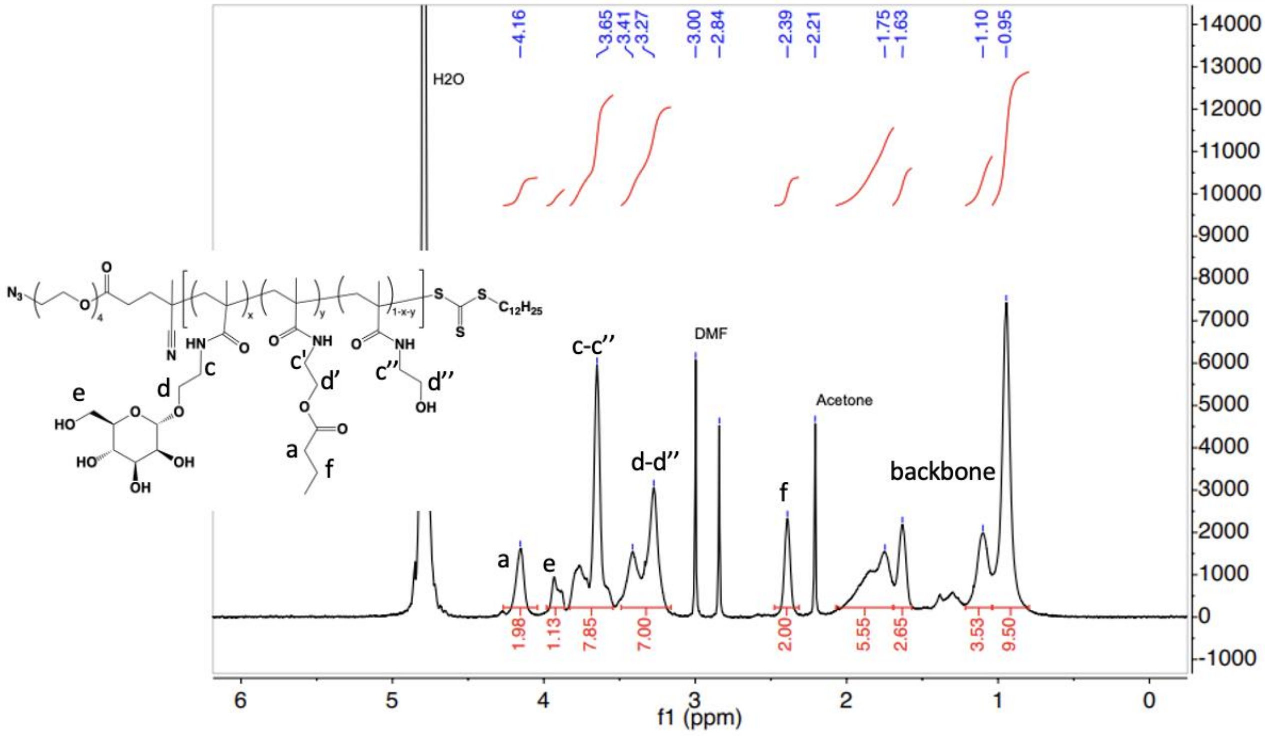

B

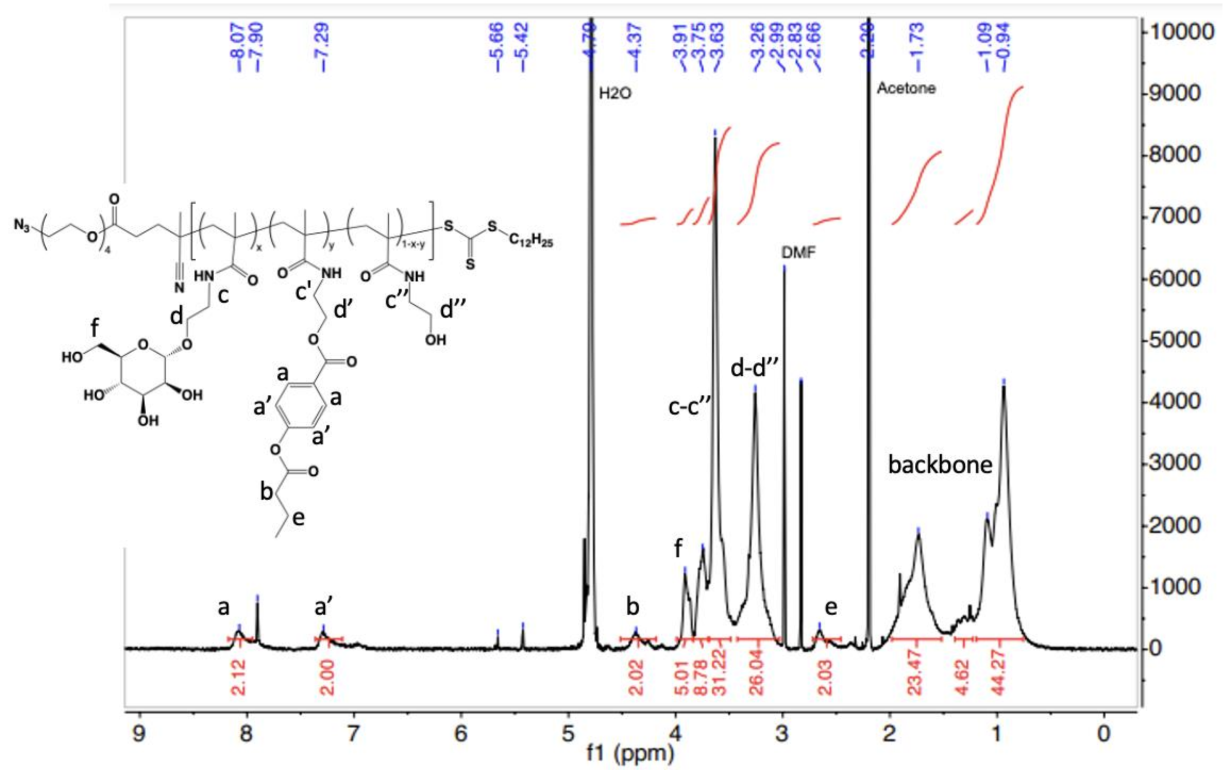

Figure S1. Proton NMR characterization of butyrate-containing copolymers. NMR spectra of purified (A) pMan-but and (B) pMan-PhBut in $CDCl_3$ using a 400 MHz spectrometer.

A **B**

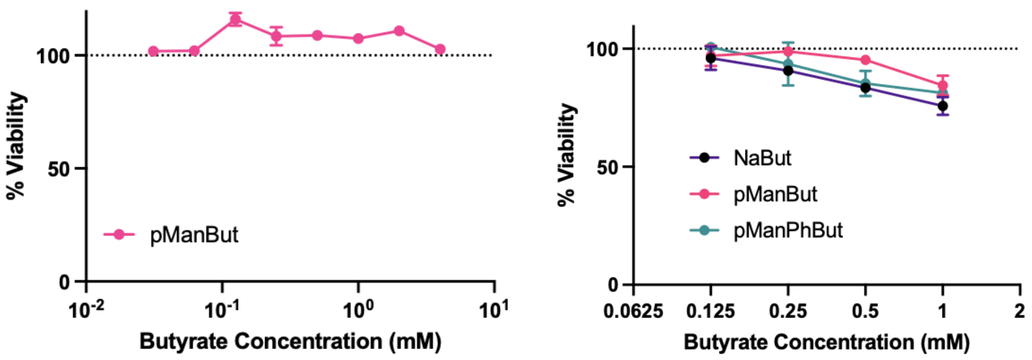

Figure S2. pMan-But and pMan-PhBut are non-toxic to mBMDCs and RAW 264.7 cells. (A) RAW 264.7 cells (n = 3) were plated and treated with pMan-But at varying concentrations. After 24 hours, cells were analyzed using MTT Cell Viability Assay (ThermoFisher) according to manufacturer's protocol. **(B)** BMDCs (n = 4) were plated and treated with butyrate constructs and LPS, as stated in Figure 2 and methods. Cells were stained with violet fixable live/dead stain (Fisher) and collected via flow cytometry. The experiment was repeated twice with similar results. For both experiments, data are plotted as mean +/- SEM.

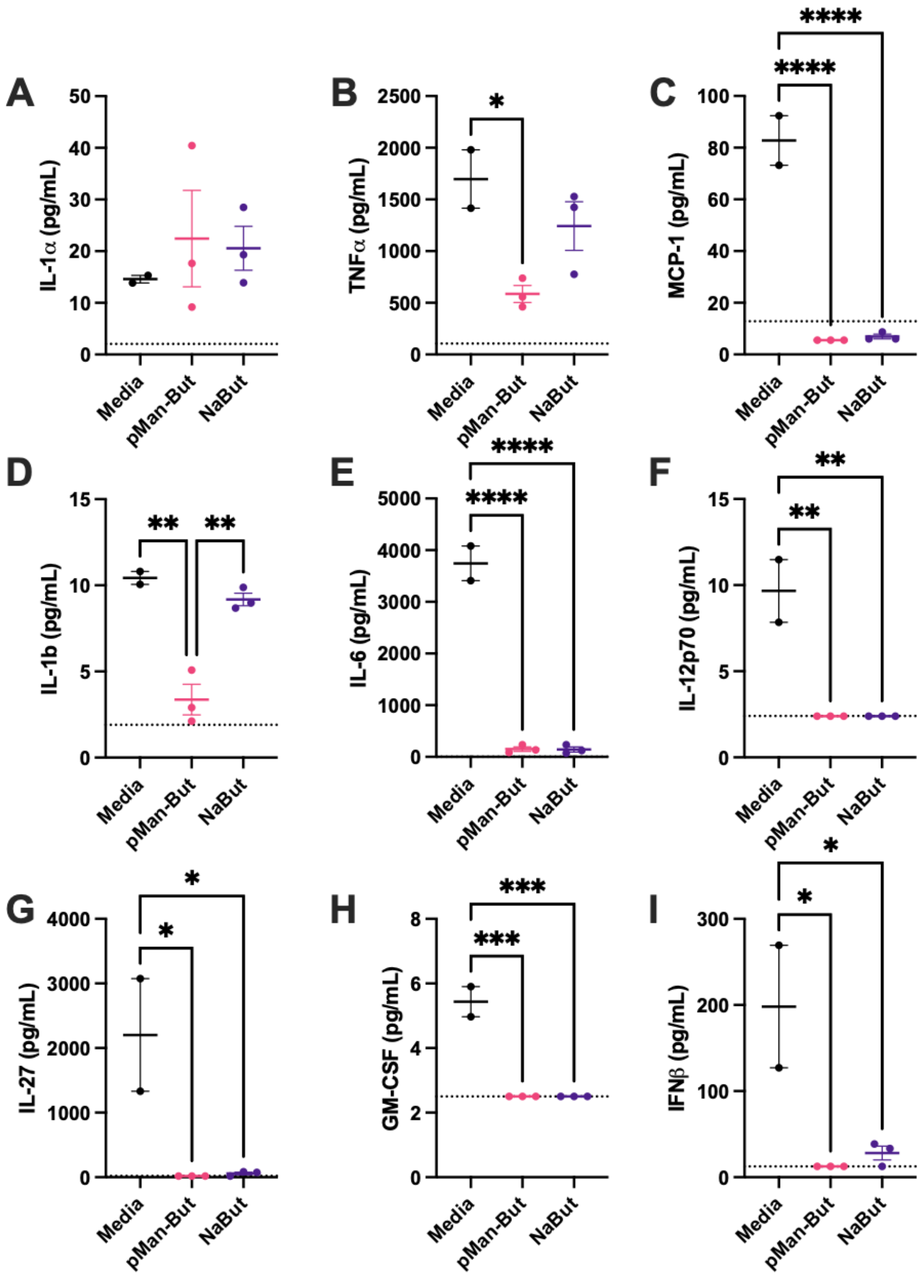

Figure S3. pMan-But suppresses pro-inflammatory cytokine and chemokine signaling from mBMDCs. The *in vitro* experiment from Figure 2 was repeated with slight differences. Briefly, cells were plated, pre-treated with 0.5 mM butyrate equivalent of pMan-But or NaBut, and, after 24 hours, challenged with LPS. The supernatant was analyzed using LegendPlex mouse inflammation panel. Suppression of pro-inflammatory cytokine and chemokine signaling was observed in all analytes except (**A**) IL-1a. These included (**B**) TNFa, (**C**) MCP-1, (**D**) IL-1b, (**E**) IL-6, (**F**) IL-12p70, (**G**) IL027, (**H**) GM-CSF, and (**I**) IFNb. Interestingly, the butyrate-induced suppression was stronger than that of free NaBut in TNFa (**B**) and (**D**) IL-1b. Statistical analysis was performed using ordinary one-way analysis of variance with multiple comparisons between each group. Data are plotted as mean +/- SEM.

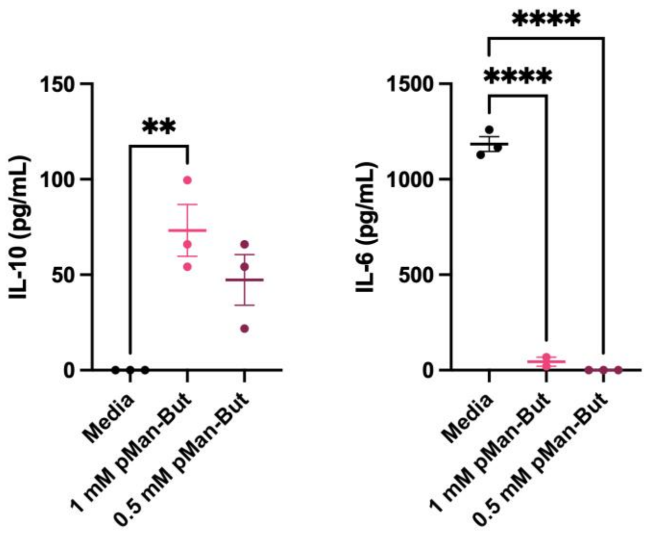

Figure S4. pMan-But alters cytokine signals from RAW 246.7 cells. The *in vitro* experiment from Figure 2 was repeated using RAW 264.7 macrophage-like cells. Briefly, cells were plated, pre-treated with two concentrations of pMan-But, and, after 24 hours, challenged with LPS. ELISA analysis of the cell culture supernatant revealed a dose-dependent increase in the anti-inflammatory cytokine IL-10 and similar dose-dependent suppression of LPS-induced, pro-inflammatory cytokine IL-6. Statistical analysis was performed using ordinary one-way analysis of variance with multiple comparisons between each group. Data are shown as mean +/- SEM. **p<0.01; ****p<0.0001.

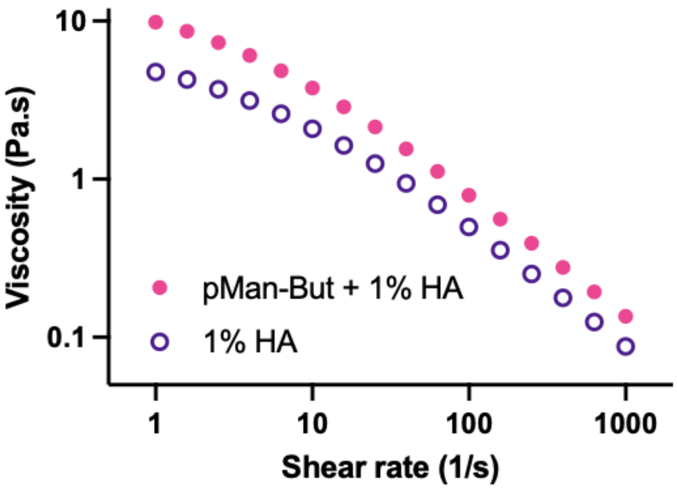

Figure S5. Polymer addition increases the viscosity of HA gel. Shear rheology characterization of pMan-But using torsional rheometry. Steady shear viscosity of 1% HA in PBS as a function of shear rate exhibits shear thinning response. Addition of pMan-But increases the low-rate viscosity with analogous degree of shear thinning behavior. Rheological measurements were conducted using a TA Instruments Discovery HR-30 shear rheometer with a smooth parallel plate geometry (d = 40 mm) using a gap size of 0.3 mm. Measurements were performed at room temperature (22 °C). Steady shear viscosity values were measurable in the shear rates in the range of $1–10^3$ 1/s.

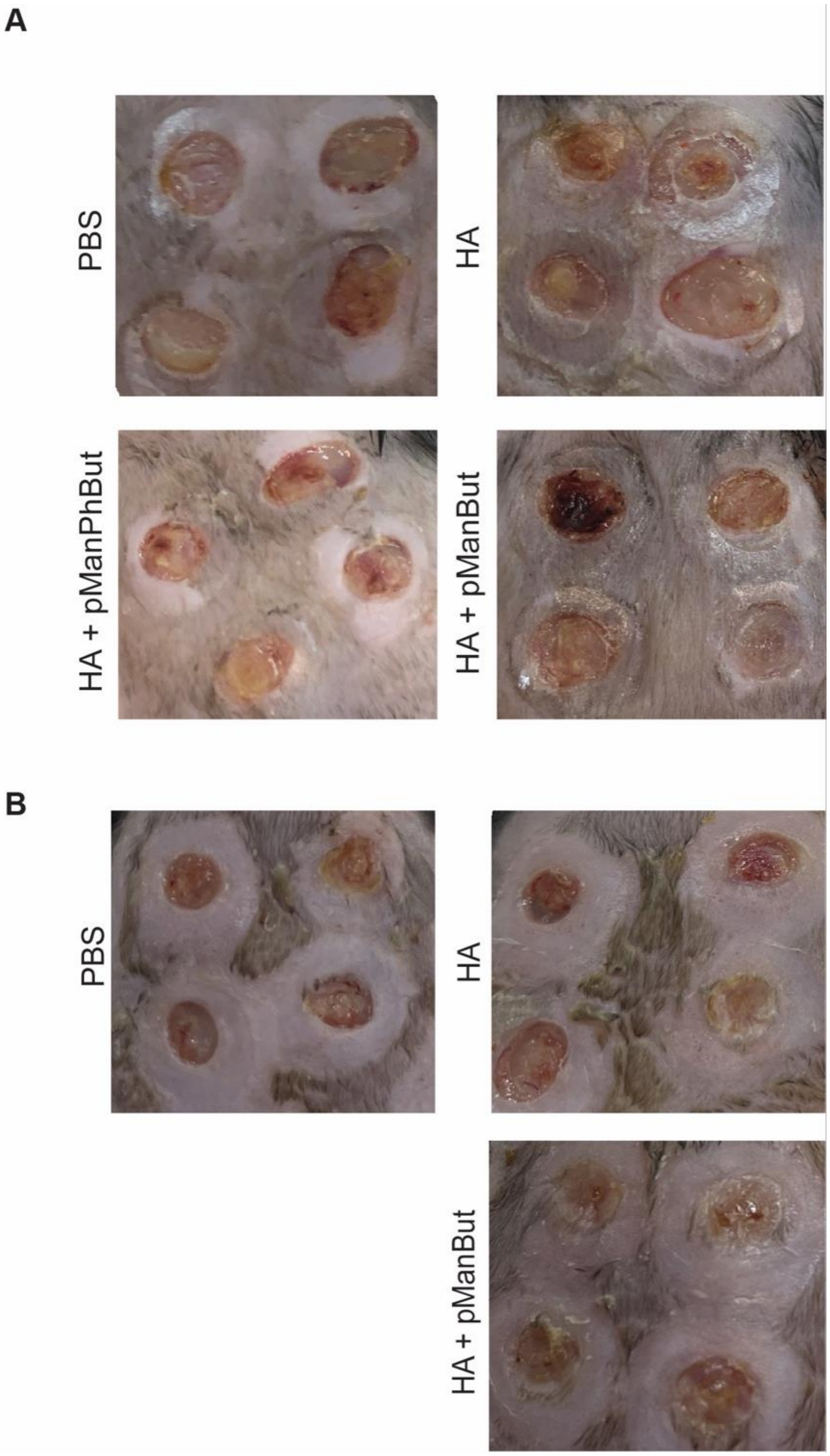

Figure S6. Wound Photographs. Endpoint photographs taken at (**A**) Day 7 and (**B**) Day 11.

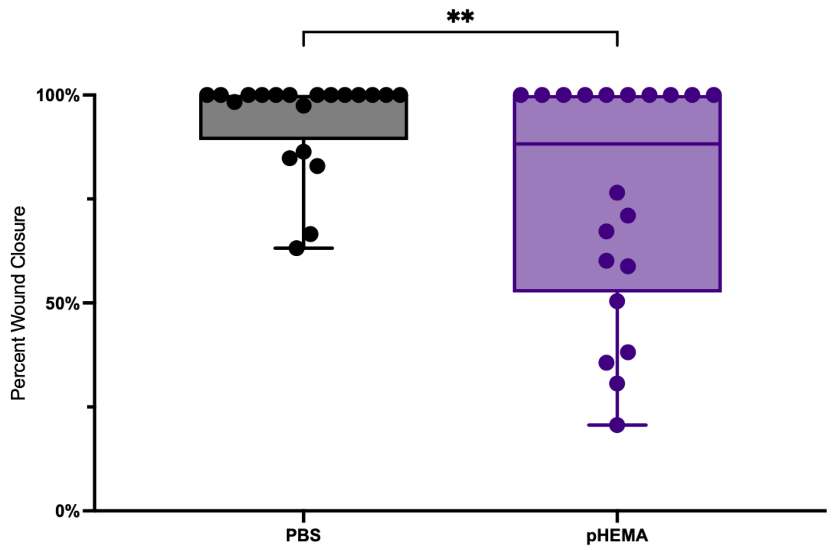

Figure S7. PBS vs. pHEMA healing efficacy. This experiment was conducted as described in Methods. The endpoint is on Day 11 post-treatment. Statistical analysis was performed using an unpaired t-test. **p<0.01

Supplementary Methods

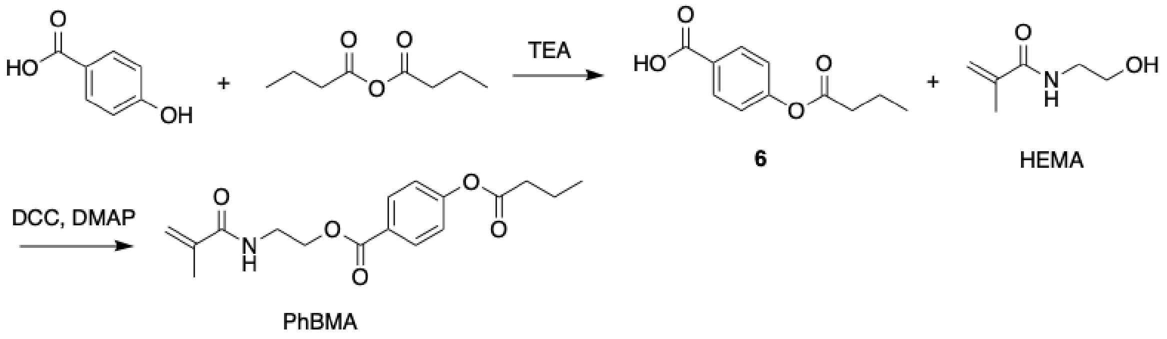

PhBMA synthesis schema

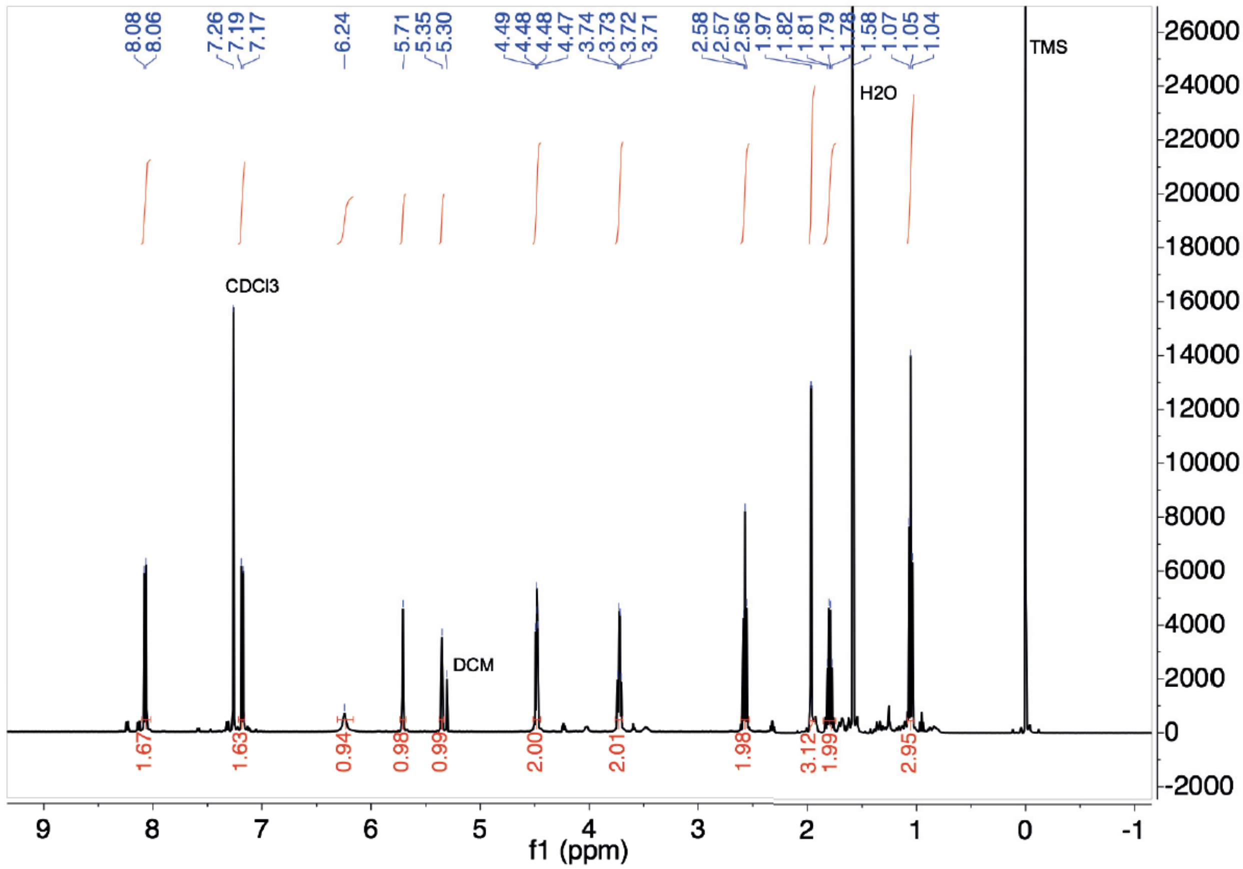

PhBMA proton NMR

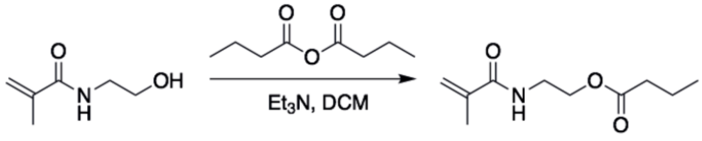

BMA synthesis schema

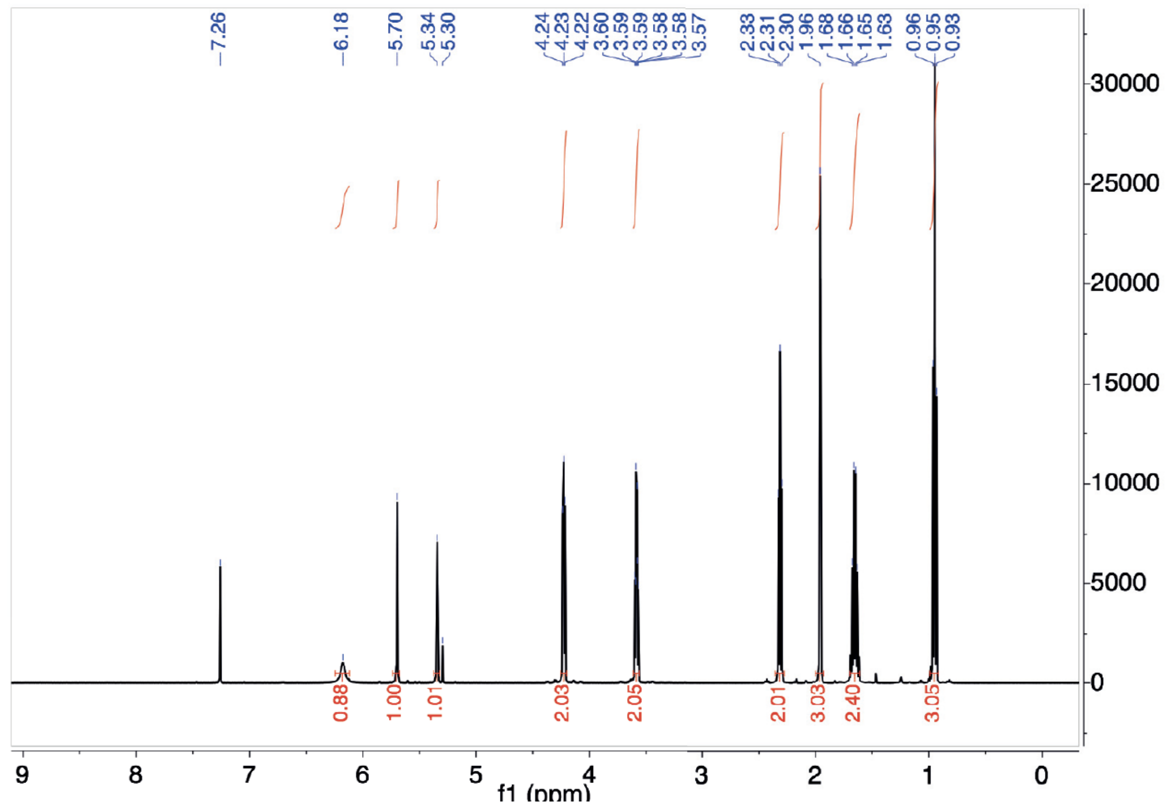

BMA proton NMR
